# Insulin at 100: still central in protein-based therapy for chronic disease

**DOI:** 10.1038/s43856-021-00004-4

**Published:** 2021-06-30

**Authors:** Peter Kurtzhals, Bernt Johan von Scholten, Frederik Flindt Kreiner, Stephen Charles Langford Gough

**Affiliations:** Global Chief Medical Office, Novo Nordisk A/S, Søborg, Denmark

**Keywords:** Diabetes, Pharmacology, Type 1 diabetes

## Abstract

Kurtzhals et al. mark the centenary of the discovery of insulin by looking back at how this model protein has changed science and medicine. They discuss how lessons learned from insulin over the last one hundred years are shaping the present and future of protein-based therapies for chronic disease.

## Teaser

The discovery of insulin has inspired several pivotal medical and scientific developments during the past 100 years. Here, we describe how insulin as a model protein will drive future advances in peptide- and protein-based therapies for chronic diseases.

At the centenary of the discovery of insulin, the medical and scientific communities pause to reflect on what the pancreatic hormone has meant not just for people with diabetes but indeed for medicine and science in general. As made clear by others^1^, insulin instantly became a life-saving therapy for people with type 1 diabetes and has played a critical role to lower blood glucose and reduce late-stage complications in type 2 diabetes. However, it is equally worthwhile to think forward and consider the many novel developments that are in store, arguably as consequences of the learnings gained with insulin over the past 100 years. Here, we do just that based on a brief review of the key insulin-related achievements in medicine and science.

## Insulin as a model protein

Insulin has played a pivotal role far beyond its application in diabetes therapy^1^. For example, insulin was the first protein to be sequenced, the first to be chemically synthesised, among the first to be measured by radio immunoassay, the first shown to be processed as a prohormone and the first protein to be produced in large quantities by genetic engineering. Recombinant genetic engineering techniques are now pivotal in the production of several biopharmaceuticals, including vaccines. Moreover, insulin analogues were the first examples of a rationally engineered protein-based therapy^2^. Analogues with tailored time-action profiles (short-acting and longer-acting insulin) were first engineered in the 1980’s and 1990’s, and designed based on the understanding of the insulin structure as revealed by Nobel Laureates Sanger and Hodgkin. The analogues had pharmacokinetic profiles that better mimicked the physiological pattern of insulin release and hence provided benefits of improved glycaemic control with reduced risk of hypoglycaemia. Subsequently, the technological foundation for amino-acid substitutions and side-chain modifications developed in connection with the creation of insulin analogues^3^ has been used to spearhead the development of other classes of peptide- and protein-based therapies where the half-life of the natural hormone is too short to allow for pharmacological use. One of the most notable examples is the glucagon-like peptide-1 (GLP-1) receptor agonist (RA) drug class, licensed for the treatment of diabetes and obesity^4^. GLP-1 is a 31-amino-acid peptide secreted from the intestinal L-cells upon food intake that has a role in glucose and energy homoeostasis. GLP-1 is of similar molecular size as the 51-amino-acid insulin protein and also has a short half-life in its native form. Technologies developed to prolong the action profile of insulin have to a large extent been applied to GLP-1 and vice versa, including both amino-acid substitutions and side-chain modifications.

The developments that enabled the introduction of longer-acting insulins for once-daily injection have also led to once-weekly GLP-1 analogues and most recently to a once-weekly insulin^5^. In addition, growth hormone replacement therapy has recently been developed for once-weekly subcutaneous administration using the same technology as for insulin and GLP-1, i.e., attachment of a fatty-acid side chain to the amino-acid backbone to confer albumin binding^6^.

Insulin is also believed to be the first protein-based therapy to be made available as a combination of products, combining two insulins with different time-action profiles^7^. This development reduced the required injection frequency and in turn inspired the development of other combination products, including the co-formulation of an insulin analogue and a GLP-1 analogue^8^. This combination therapy provides a more effective lowering of blood glucose with a low risk of hypoglycaemia and little or no weight gain in people with type 2 diabetes compared with insulin alone. More recently, dual or triple agonists, where peptide- or protein-based molecules are engineered to activate more than one receptor, have been designed for the treatment of diabetes or obesity. Examples of such compounds in clinical development include dual agonists targeting the GLP-1 and glucagon receptors, dual agonists targeting the receptors of GLP-1 and gastric inhibitory polypeptide (GIP) and triple agonists targeting these three receptors (GLP-1/glucagon/GIP)^9^. GIP is a 42-amino-acid peptide secreted from intestinal K cells, and like GLP-1, it is an incretin hormone that as its primary action increases the insulin response to an oral glucose load. These molecules can lead the way in a future where polypharmacology may be required for effective treatment and sustained benefits. For example, in weight management, it is generally recognised that combination therapy will be required for continued weight loss. Similarly, in type 2 diabetes, which is a gradually progressing disease, combination therapies will be needed to control glycaemia over time and to address the various clinical aspects of the multifactorial metabolic syndrome.

Technologies developed for insulin, such as the use of absorption enhancers, paved the way for the ground-breaking development and approval of the first-ever large peptide-based drug, semaglutide, delivered in a tablet for systemic exposure.

## Insulin’s discovery is shaping the future

Looking further ahead, the combination of molecular engineering and delivery technology will likely be crucial in advancing peptide- or protein-based therapies for diabetes and other chronic conditions. Spearheading the development of protein delivery systems, in 1985 insulin was the first drug for which pen-shaped injectors were introduced. Future developments in this area will also be centred around insulin and be assisted by automatisation and digitalisation. Insulin pumps coupled with glucose-monitoring systems are being developed to allow for automated insulin delivery in response to the ambient blood glucose level^10^. Over time, data from the pump and glucose monitor may, via algorithms trained by artificial intelligence, allow for a fully closed-loop artificial pancreas. Intelligent injection pens with data connectivity features will likely be made available to memorise and guide the time and size of the given doses. Such devices will likely be useful to inform the dialogue between patient and physician with regards to adherence and control, the lack of which remains challenging even with today’s efficacious and safe diabetes medicines. Although initially developed for insulin, these delivery technologies are expectedly applicable for a range of other protein-based therapies.

At last, a holy grail in peptide- or protein-based therapy delivery has been the design of oral medications. Insulin has been at the centre of the majority of such research in both industry and academia. The challenge has been to overcome several barriers in a single delivery system, including the variability associated with food interaction, low bioavailability owing to cleavage by digestive enzymes, and limited passage of large molecules over the stomach or intestinal wall. Whilst successful oral delivery of long-acting insulin has actually been accomplished, bioavailability was too low for further development^11^. However, technologies developed for insulin, such as the use of absorption enhancers, paved the way for the ground-breaking development and approval of the first-ever large peptide-based drug, semaglutide, delivered in a tablet for systemic exposure^12^. For orally administered semaglutide, the co-formulated absorption enhancer facilitates the transcellular absorption of semaglutide predominantly from the stomach, further enabled by the engineered molecular properties of semaglutide, including high aqueous solubility, relatively small molecular size and a very long half-life in the circulation. This development has contributed to opening for research into alternative, technologically advanced delivery systems of potential applicability for oral delivery of a wide range of peptide- or protein-based therapies, including rather large molecules, with insulin as the model compound^13^.

The extrapolation of the technology platforms developed for insulin to GLP-1 and other peptide drugs, for example, analogues of GIP and of amylin, a 37-amino-acid peptide co-secreted with insulin from pancreatic beta cells, provide an important example of how the discovery of insulin indirectly has impacted modern medicine beyond providing a life-saving replacement therapy for people with type 1 diabetes. A side-chain-modified dual-acting GLP-1/GIP molecule is currently in development for diabetes^14^, and a side-chain-modified analogue of amylin is being investigated for the treatment of obesity (ClinicalTrials.gov ID NCT03600480). Hitherto, insulin-related technology platforms have enabled GLP-1-based treatment of type 2 diabetes and obesity, whereas the application is adjacent chronic diseases are being explored in late-stage clinical trials, for example in cardiovascular disease (ClinicalTrials.gov ID NCT03574597), chronic kidney disease (ClinicalTrials.gov ID NCT03819153) and non-alcoholic steatohepatitis (NASH; ClinicalTrials.gov ID NCT04822181).

Intriguingly, GLP-1 analogues in combination with a monoclonal anti-interleukin-21 antibody may, in turn, be useful to delay disease progression of type 1 diabetes when therapy is initiated shortly after diagnosis^15^. Thus, GLP-1-based therapies, which as outlined above were developed using insulin-inspired technologies and which originally were introduced to help people with type 2 diabetes, may also be valuable in type 1 diabetes. This indeed represents the near completion of the type 1 diabetes innovation circle that was initiated a hundred years ago. The final turn to complete this circle may be the transplantation of stem-cell-derived islets successfully differentiated and encapsulated to escape graft rejection. Clinical studies with cell-replacement therapies have been initiated by academic institutions (for example, Clinicaltrials.gov ID NCT04061746), life science companies (for example, Clinicaltrials.gov IDs NCT04786262 and NCT04678557), and a clinical proof-of-concept demonstrating restoration of endogenous insulin production could therefore become a reality in less than a decade.

## Conclusion

As described above, insulin remains a hormone with a rich heritage and a promising future. During the past century, insulin arguably has been the most influential hormone, driving seminal advancements in protein science and in medicine. It is reasonable to expect that many of the still-to-come improvements in the treatment of diabetes and other chronic diseases will build upon the learnings of the past 100 years, starting with the discovery made by four gentlemen in Toronto in 1921.
